# Body Structural Description Impairment in Complex Regional Pain Syndrome Type I

**DOI:** 10.3389/fpsyg.2022.853641

**Published:** 2022-06-09

**Authors:** Iftah Biran, Andrea Book, Liron Aviram, Noa Bregman, Einat Bahagali, Assaf Tripto

**Affiliations:** ^1^Neurological Institute, Tel Aviv Medical Center, Tel Aviv-Yafo, Israel; ^2^Division of Psychiatry, Tel Aviv Medical Center, Tel Aviv-Yafo, Israel; ^3^The Rehabilitation Hospital, Chaim Sheba Medical Center, Ramat Gan, Israel; ^4^Department of Psychology, Ari’el University, Ari’el, Israel; ^5^Department of Psychology, The Academic College of Tel Aviv – Yaffo, Tel Aviv-Yafo, Israel; ^6^Sackler School of Medicine, Tel Aviv University, Tel Aviv-Yafo, Israel; ^7^Be’eri Clinic, Clalit Health Services, Bnei Brak, Israel

**Keywords:** complex regional pain disorder, somatic symptom and related disorders, body structural description, Pain, Wechsler adult intelligence scale

## Abstract

**Background:**

Complex Regional Pain Syndrome (CRPS) is a clinical syndrome composed of chronic pain, motor impairment, and autonomic dysfunction, usually affecting a limb. Although CRPS seems to be a peripheral disorder, it is accompanied by parietal alterations leading to body schema impairments (the online representations of the body). Impairments to body structural description (the topographical bodily map) were not assessed systematically in CRPS. A patient we encountered with severe disruption to her bodily structural description led us to study this domain further.

**Aims:**

To document aberrant body structural description in subjects with CRPS using an object assembly task.

**Methods:**

Body Schema Study: 6 subjects with CRPS-I and six age and sex-matched healthy controls completed visual puzzles taken from WAIS-III and WAIS-R. The puzzles were either related to the human body or non-human body objects. Mann–Whitney U-tests were performed to compare groups’ performances.

**Results:**

The CRPS group received relatively lower scores compared to controls for human body objects (*u* = 3, *p* < 0.05), whereas the non-human object scoring did not reveal significant differences between groups (*u* = 9, *p* > 0.05).

**Conclusion:**

CRPS subjects suffer from impaired body structural description, taking the form of body parts disassembly and body parts discontinuity. This impairment can serve as a nidus for aberrant psychological representation of the body.

## Introduction

Complex Regional Pain Syndrome (CRPS) is a chronic neuropathic pain syndrome usually involves one limb. In most cases, it occurs after an injury to the affected limb. It is subdivided into two types—Type I, in which there is no significant nerve damage, and type II, which involves nerve damage. The clinical presentation is composed of a triad of (i) Chronic pain, usually burning in its nature, accompanied by increased sensitivity to pain (hyperalgesia); (ii) Motor impairment with a decrease in motor function of the limb leading to contractures and deformations and (iii) Autonomic dysfunction (edema, sweating, change in color and temperature, atrophic changes; Wilson and Serpell, 2007; Ott and Maihofner, 2018). The condition can be devastating to the extent that some patients seek and, at times, get their affected limb amputated (Dielissen et al., 1995).

The pathophysiology of CRPS involves various peripheral mechanisms (i.e., neurogenic inflammation and peripheral sensitization). However, these peripheral mechanisms cannot explain all the signs related to CRPS (Reinersmann et al., 2013). Nonetheless, CRPS seems to be *prima facie* a peripheral disorder. Accordingly, peripheral dysfunction is regarded as the leading etiological and pathogenic factor. However, there is also a role for central nervous system pathology in general and cortical dysfunction in particular, presumably involving the parietal cortices. There are changes in the cortical sensory parietal representations of the affected limb as documented in fMRI and magnetoencephalographic studies. Sensory fields related to the affected limb are diminished and return to their original size upon recovery (Schwoebel et al., 2001; Schwenkreis et al., 2009; Swart et al., 2009; Strauss et al., 2021). However, this was recently debated. For an alternative view, see (Mancini et al., 2019).

CRPS patients often report distortion in body perception in general and in the perception of the impaired limb in particular. Patients report feeling of being detached and dissociated from the affected body part, the loss of specific anatomical parts, and the inability to mentally visualize segments of the affected body parts (Lewis et al., 2007, 2010; Lewis and McCabe, 2010; Lewis and Schweinhardt, 2012). This could be related to the impairment of the neurological apparatus related to body representations. Body representations encompass three major types: (i) Body image, which is the semantic knowledge related to the body (name of body parts, their use, corresponding objects used with these body parts), (ii) Body schema, which entails a dynamic representation of the human body that is based on body localization in space and (iii) Body structural description which is a topographical map of the human body (e.g., the continuity of body parts; which body part is connected to which?; Sirigu et al., 1991; Coslett, 1998; Buxbaum and Coslett, 2001; Di Vita et al., 2020).

Most studies on the deficits of body representations in CRPS looked at body schema. This function can be studied through mental rotation tasks involving the affected limb (handedness tasks). In these tasks, the subject must judge the laterality of a limb presented from different angles, either from its back or its front. Subjects with CRPS have prolonged reaction times in this task correlated with the parietal dysfunction (Schwoebel et al., 2001, 2002). Other studies looked at body schema impairment by studying the sensation of limb position and limb movement (Brun et al., 2019).

To the best of our knowledge, the body structural description was not studied systematically in CRPS (Echalier et al., 2020; Halicka et al., 2020). It is at times documented in patients’ narratives as a missing part of a limb or a “void” in a limb (Lewis et al., 2007; Lewis and McCabe, 2010) or as a disconnected body part (Galer et al., 1995). A few studies documented impaired finger recognition which might be related to impaired structural description (Förderreuther et al., 2004; Cohen et al., 2013; Kuttikat et al., 2018).

The above observations can be related to the unclear demarcation between CRPS and conversion disorders at times. While in CRPS, there is usually a clear physical antecedent and clear signs of physical signs, this is not the case with conversion disorder. However, in both diseases, the symptoms and signs are not fully explained by a biological physical mechanism, there is an association with psychiatric comorbidities, such as post-traumatic stress disorder, somatization, and depression, and both can follow a physical trauma (Shiri et al., 2003; Grande et al., 2004).

We encountered a subject with CRPS who presented with severe alterations in her body structural description. This female patient has a long-standing history of verbal and physical abuse and ambivalence toward others. She presented with right leg CRPS following a minor fall and ankle sprain. She was treated with physiotherapy, occupational therapy, and weekly psychoanalytic-oriented psychotherapy. In treatment, she raised what seemed to be an adequate concern with the symptomatic limb describing anxiety related to the pain, the obscure disease process, and the accompanying dysfunction.

On the other hand, following an appearance of a callous bleeding wound on the affected leg, she raised peculiar and bizarre misrepresentations related to the leg. This took the form of a profound distortion of the structural description of her lower body. The bleeding callous became a bleeding vulva dentata with teeth and animals running within (Arlow, 1955). This propagated to the entire leg that became an emblem of a huge penis–vagina–rectum complex resembling a defecating, menstruating cloaca suggesting a regression to primitive sexuality (Freud, 1905). To the best of our knowledge, no similar cases of CRPS with similar sexual cathexis to the affected limb were previously reported. However, a previous report suggested profuse cathexis of emotional substantial feelings and self-parts onto the affected limb (Zarnegar, 2015). In any case, as suggested, patients with CRPS are reluctant to report their bizarre ideations toward the affected body parts. This might lead to similar reports’ Sparsity (Lewis and McCabe, 2010).

In this study, we wanted to assess similar body structural fragmentation in CRPS. Our patient’s bizarre presentation might have been an outlier of less dramatic and more common presentations related to the impairment of body structural description. We hypothesized that patients with CRPS might show a similar albeit less profound deficit in body structural description and would be more impaired than controls with tasks requiring the assembly of human body parts suggesting a deficit in the body’s structural description. This could shed light on the extent of the functional and cognitive anomalies related to the body found in CRPS and contribute to developing specific rehabilitation interventions that address these deficits (i.e., body structural description) specifically. To test this, we administered the object assembly subtest items of the WAIS-III and the WAIS-R (Wechsler, 1981, 1997). These subtests include puzzles of ordinary objects, and participants are required to connect pieces of puzzles into meaningful objects. We compared the performance on human body objects Assembly (HBOA; hand, human profile, and human figure) with that on non-human body objects Assembly (Non-HBOA; house, butterfly, and elephant). As mentioned above, we expected CRPS subjects to be relatively more impaired with the assembly of human body objects than with the assembly of non-human body objects.

## Materials and Methods

### Subjects

Six subjects with CRPS (type I) and six age and sex-matched healthy controls. Subjects with CRPS were recruited from the outpatient rehabilitation service at Chaim Sheba Medical Center. Control subjects were recruited from hospital and university employees. Inclusion criteria for the CRPS subjects were age > 18 years and CRPS of one limb. Criteria for the control group were age > 18 years and matched with the study group according to sex, age, and education. Exclusion criteria for CRPS and control groups comprised having another pain syndrome, having an orthopedic injury, documented head injury, or inability to perform tasks due to language or physical injury limitations. Control subjects with previously recorded psychiatric disorders were excluded.

Subjects completed demographic information questionnaires (age, family status, education, occupation). Subjects also completed the following questionnaires:

1. Mental Health Inventory (MHI; Veit and Ware, 1983; Florian and Drori, 1990) contains 38 self-reported items designated to assess patients’ mental health during the previous month. Items can be used to evaluate a total mental health index, made up of two global scales (psychological wellbeing and psychological distress) and six subscales (anxiety, depression, loss of behavioral or emotional control, general positive affect, emotional ties, and life satisfaction). A higher Global MHI score indicates better psychological wellbeing.
2. The MOS 36-item short-form health survey (SF-36; Ware and Sherbourne, 1992). This survey consists of 36 self-reported items designated to assess patients’ health status on eight subscales (pain, general health perception, emotional wellbeing, physical function, role limitation due to physical health, role limitation due to emotional health, social function, and fatigue\energy). We used the pain, emotional health, and general health subscales. Scoring was determined by the sum of items. A lower score indicates a more severe disease.
3. The Bath CRPS Body Perception Disturbance Scale comprises seven self-reported items and assesses changes in patients’ perception of an affected limb. A higher score denotes more disturbance, with 57 being the maximum total score (Lewis and McCabe, 2010). The Questionnaire was administered only to the CRPS group.
4. Assessment of Pain Severity—Pain on the day of the testing was assessed using a Visual Analog Scale (VAS) of zero to ten taken from the McGill Pain Questionnaire (Waldman, 2009). Pain in the last 4 weeks before testing was assessed using items 22–23 from SF-36 (Ware and Sherbourne, 1992).
5. Assessment of PTSD symptomatology—Patients completed the PTSD Inventory. This 17 items questionnaire assesses the severity of post-traumatic stress disorder symptomatology. A cutoff score of 50 and above is recommended for a probable diagnosis of PTSD (Solomon et al., 1993; Weathers et al., 1993; Ginzburg et al., 2002).

### The Experimental Task—Object Assembly Task

Six visual puzzles taken from WAIS-III and WAIS-R (Wechsler, 1981, 1997). The puzzles were human body objects [hand, human profile, and human figure] or non-human body objects [house, butterfly, and elephant]. Scoring for each item was determined by the number of correct connections and performance times according to the WAIS-III and WAIS-R manuals (Wechsler, 1981, 1997). We calculated the average score for each category. The higher the score, the better the performance is (Human Body Objects: 0–10, Non-Human Body Objects: 0–10.6, All objects: 0–10.33).

### Ethics

The study was approved by the ethical committee of Chaim Sheba Medical Center and following the Helsinki declaration. All subjects were explained the study protocol and gave written informed consent.

### Statistics

Due to the small number of participants and non-normal distribution of both the experimental and demographic data as visualized in histograms, Mann–Whitney U-tests were performed to compare CRPS and control group performances and Spearman Analysis to study correlations. As we calculated three comparisons of the experimental task, we used a Bonferroni post-hoc analysis correction and multiplied the calculated value of p by three. We used IBM SPSS Statistics, version 27. When appropriate, we report the average and standard deviation. We used the “ggridges” R-Package for the rain cloud plots (Jeehyoung, 2022).

## Results

### Demographic Data

Each group included four women and two men. Between-group comparison was conducted using Mann–Whitney U-tests to assure appropriate matching. There were no significant group differences in age (Controls 31.2 ± 13.40 years, CRPS 31.0 ± 12.41 years, *U* = 18, *p* > 0.05) or education (Controls 14.17 ± 3.54 years, CRPS 14.17 ± 3.13 years, *U* = 18, p > 0.05).

### Clinical Characteristics

Controls reported better general health compared with CRPS patients (SF-36 General Health: Controls 95.8 ± 5.8, CRPS 55.0 ± 32.0, *U* = 4.5, *p* = 0.026), had less pain (SF-36 Pain: Controls 94.68 ± 9.3, CRPS 26.3 ± 31.3, *U* = 1, *p* = 0.006; Pain-VAS-Controls 0.67 ± 1.6, CRPS 6.2 ± 3.1, *U* = 1, *p* = 0.009), better mental health (Global MHI score: Controls 183.7 ± 23.6, CRPS 119.8 ± 41.0, U = 2, *p* = 0.010) and better emotional functioning (SF-36 Emotional: Controls 82.7 ± 9.4, CRPS 46.0 ± 20.4, *U* = 2.5, *p* = 0.012). CRPS patients scored 29.3 ± 14.4 on the BATH scale suggesting a relative impairment in their body perception (Lewis and Schweinhardt, 2012). All CRPS patients scored on the PTSD inventory below the cutoff (18.2 ± 8.4, cutoff = 50; Weathers et al., 1993, see Tables 1, 2).

**Figure 1:**
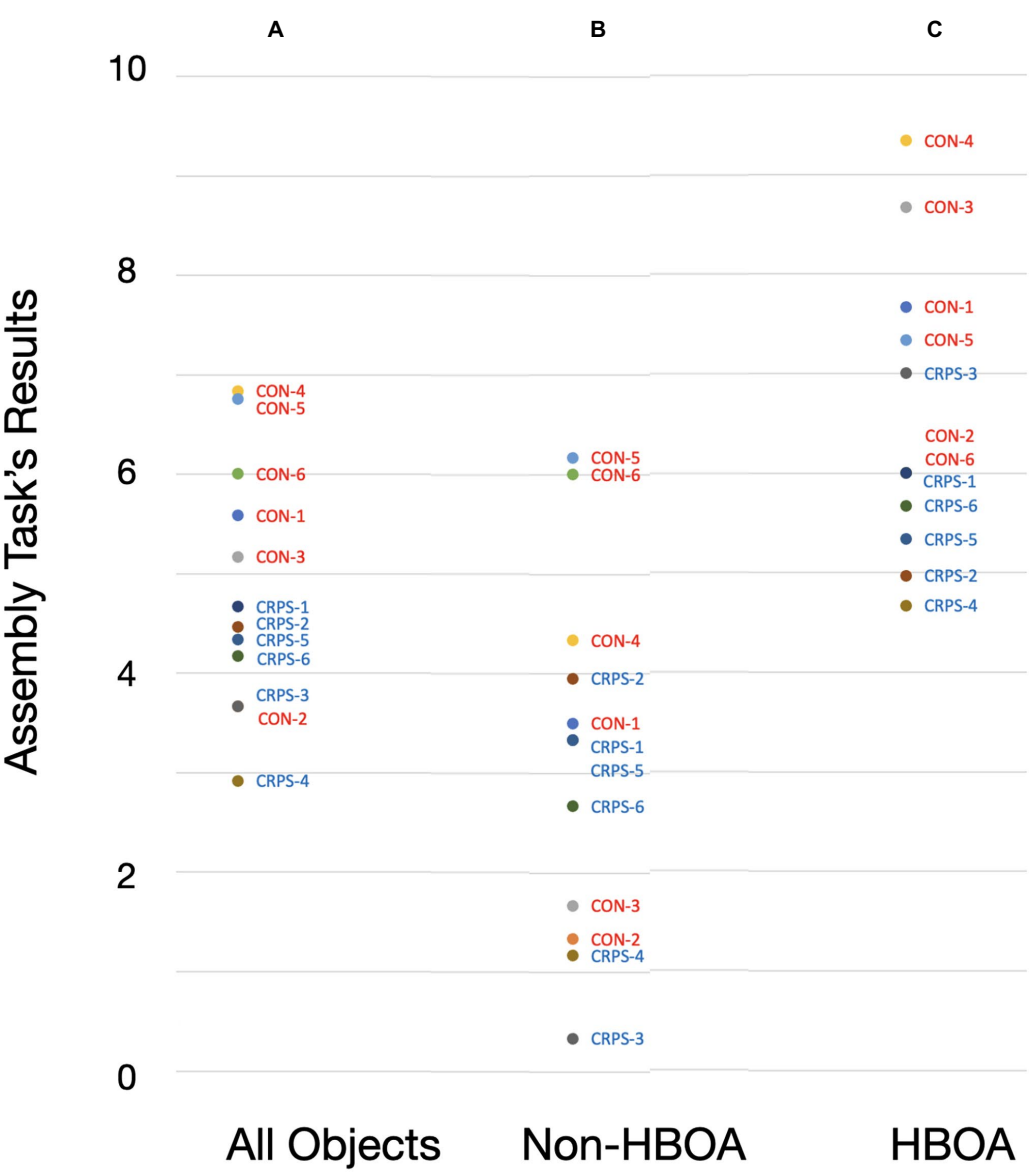
Assembly tasks’ resutls by subjects. A—Performance on all stimuli (All Objects); B—Performance on Non-Human Body Object Assembly (Non-HBOA); C—Performance on Human Body Object Assembly (HBOA).

**Table 1:** Demographic and Clinical Characteristics of the CRPS Patients.

	Sex	Age range (Years)	Education range (Years)	Body part	Meds	Psychiatric diagnosis	SF-36 general health	SF-36 Pain	SF-36 emotional	Pain (VAS 0–10)	Global MHI score	Bath score	PTSD inventory
1	M	35–50	13–20	LE	P,BDZ	None	65	45	44	5	113	31	30
2	F	20–35	8–12	UE	O,P,S	None	25	0	24	8	67	38	25
3	F	50–65	8–12	LE	TCA,O,CB	None	80	10	44	9	139	51	21
4	M	20–35	13–20	UE	SN,BDZ,CB	DEP	20	0	28	9	81	19	12
5	F	20–35	8–12	LE	P,O	ADHD	40	22.5	80	5	176	10	11
6	F	20–35	13–20	UE	P,CB	None	100	80	56	1	143	27	10
Average (SD)	–	31 (12.41)	14.17 (3.13)	–	–	–	55.0 (32.0)	26.3 (31.3)	82.7 (9.4)	6.2 (3.1)	119.8 (41.0)	29.3 (14.4)	18.2 (8.4)

*Body Part: LE, Lower Extremity, UE; Upper Extremity; Meds, Medications; TCA, Tricyclic Anti-Depressants; BDZ, Benzodiazepines; CB, Cannabis; O, Opiates; P, Pregabalin; S, SSRIs; SN, SNRIs; SD, Standard Deviation. Age and education are given in range to mask subjects’ identity further*.

**Table 2:** Demographic Data, Clinical Characteristics and Experimental Task Results.

	Controls	CRPS	
*Demographics*			
No (F/M)	6 (4/2)	6 (4/2)	
Age (SD)	31.2 (13.4)	31.0 (12.4)	*U* = 18, *p* = 1
Education (SD)	14.2 (3.5)	14.2 (3.1)	*U* = 18, *p* = 1
*Clinical Characteristics*			
SF-36 General Health (SD)	95.8 (5.8)	55.0 (32.0)	*U* = 4.5, *p* = 0.027
SF-36 Pain (SD)	94.6 (9.3)	26.3 (31.3)	*U* = 1, *p* = 0.006
SF-36 Emotional (SD)	82.7 (9.4)	46.0 (20.4)	*U* = 2.5, *p* = 0.012
Global MHI score (SD)	183.7 (23.6)	119.8 (41.0)	*U* = 2, *p* = 0.010
Current Pain Severity (SD)	0.7 (1.6)	6.2 (3.1)	*U* = 1, *p* = 0.009
Bath score (SD)	–	29.3 (14.4)	–
PTSD Inventory (SD)	–	18.2 (8.4)	–
*Experimental Task*			
All Objects (SD)	5.7 (1.2)	4.0 (0.6)	*U* = 4.5, *p* = 0.090
HBOA (SD)	7.5 (1.4)	5.6 (0.8)	*U* = 3, *p* = 0.048
Non-HBOA (SD)	3.8 (2.1)	2.5 (1.4)	*U* = 9, *p* = 0.447

*HBOA, Human Body Object Assembly; Non-HBOA, Non-Human Body Object Assembly. SD, Standard Deviation*.

### Experimental Task

**Figure 2:**
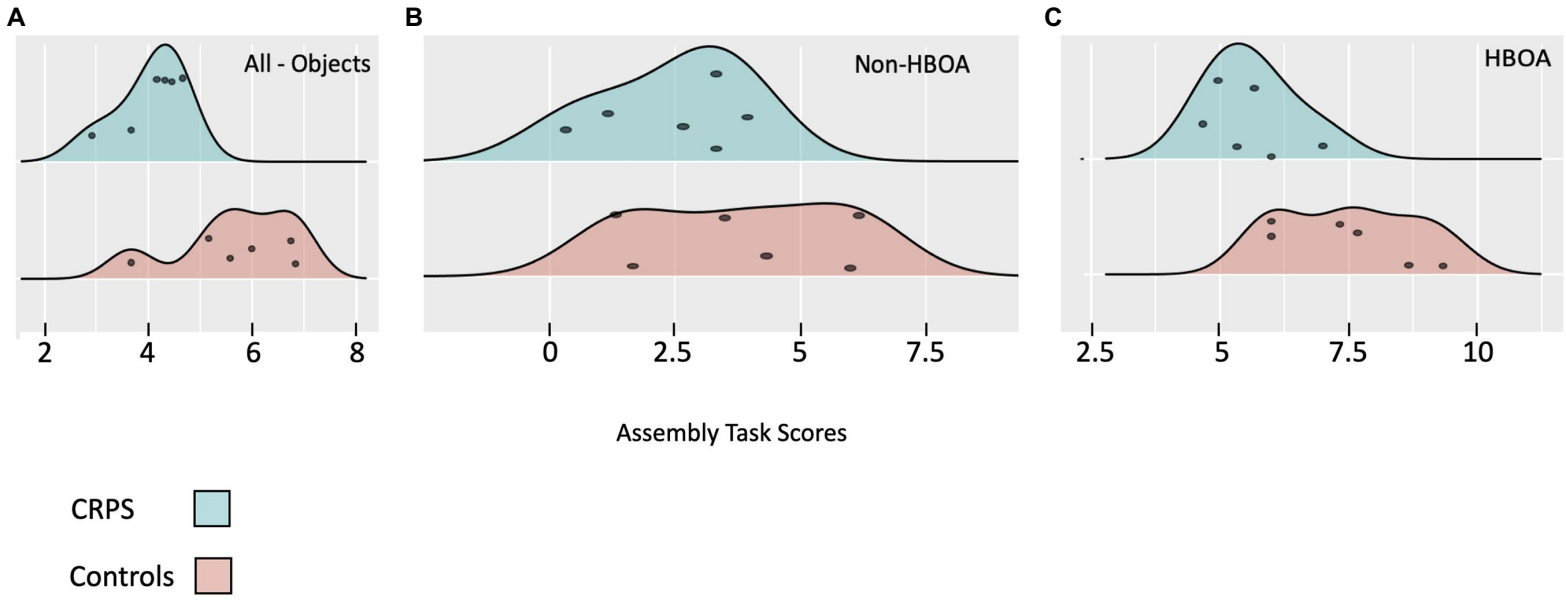
Performance on the Assembly Task presented in a rain cloud plot. **Panel A**—results for all objects; **Panel B**—Results for the Non-Human Body Objects Assembly (Non-HBOA); **Panel C**—Results for the Human Body Objects Assembly (HBOA).

Mann–Whitney U-tests compared CRPS and control group performance on the object assembly task. We conducted three comparisons. The first comparison was the total score for all six objects; the second compared Human Body Objects Assembly (HBOA), and the third compared Non-Human Body Objects Assembly (Non-HBOA). A significant difference was found only for the HBOA (Controls 7.5 ± 1.45.6 ± 0.8, CRPS 5.6 ± 0.8, u = 3, *p* = 0.048) but not for the total score for all assembly objects (Controls 5.7 ± 1.2, CRPS 4.0 ± 0.6, u = 4.5, *p* = 0.090) and not for the Non-HBOA (Controls 3.8 ± 2.1, CRPS 2.5 ± 1.4, u = 9, *p* = 0.447, see Table 2; Figures 1, 2).

Spearman correlations were performed to determine the relationship between performance on the object assembly tasks and pain levels, psychological wellbeing, general health, and levels of body perception disturbances (Bath Scale). There were strong and significant correlations between the performance on HBOA and current pain (*r*_s_ = − 0.660, *p* = 0.027), SF-36 Pain (*r*_s_ = 0.707, *p* = 0.010), and SF-36 General Health (*r*_s_ = 0.582, *p* = 0.047). There were no significant correlations for Non-HBOA. There were strong and significant correlation between the performance on all objects and SF-36 Pain (*r*_s_ = 0.704, *p* = 0.011) and SF-36 Emotional (*r*_s_ = 0.617, *p* = 0.032, see Table 3).

**Table 3:** Spearman Correlations between The Object Assembly Tasks and The Demographic and Clinical Data.

		Age	Education	MHI total	Pain (VAS)	SF-36 Pain	SF-36 General health	SF-36 Emotional	Bath scale
HBOA	Spearman	0.254	−0.030	0.563	−0.660^*^	0.707^*^	0.582^*^	0.539	0.543
	Sig	0.425	0.926	0.056	0.027	0.010	0.047	0.071	0.266
Non-HBOA	Spearman	−0.037	0.140	0.424	−0.515	0.475	0.150	0.490	−0.116
	*p*	0.909	0.663	0.170	0.105	0.119	0.642	0.106	0.827
All Objects	Spearman	0.118	0.191	0.571	−0.586	0.704^*^	0.345	0.617^*^	0.143
	*p*	0.716	0.552	0.053	0.058	0.011	0.273	0.032	0.787

(All Objects, Human Body Object Assembly (HBOA), Non-Human Body Object Assembly (non-HBOA)) and the demographic and clinical data. (Sig. = Significance (2-tailed), ^*^ = Correlation is significant at the 0.05 level (2-tailed)).

## Discussion

We compared the performance of 6 subjects with CRPS with that of six age and sex-matched healthy controls in an object assembly task. CRPS subjects were relatively more impaired with HBOA than Non-HBOA compared with controls, suggesting a specific impairment to body structural description. This was further supported by the strong and significant correlations between performance on HBOA and pain severity and impaired health (SF-36 pain, Pain (VAS), SF-36 General Health) and the lack of correlation between performance on Non-HBOA and the clinical measures. These correlations are in accordance with our hypothesis as they suggest that the higher the pain and the health difficulties, the worst the relative impairment to body structural description.

The patients had an aberrant perception of their affected limb, as demonstrated by their score on the Bath scale, similar to what was previously reported in CRPS patients (Lewis and Schweinhardt, 2012).

The performance on the HBO suggests that the impairment is not confined to the patients’ own body but is somewhat generalized to the body of others or at least to the representations of others, as in the assembly task. In this regard, the deficit of the CRPS patients could involve one’s own body (“Autotopagnosia”) or the body of others (“Heterotopagnosia”). Following Gerstmann, this could be called “Somatotopagnosia,” denoting an impairment in locating body parts regardless of the body’s ownership (one’s own or bodies of others; Gerstmann, 1942).

Our findings shed new light on the body representations in CRPS and point to a discrete body structural description deficit. However, they are in line with previous observations of impaired body structural description in other neurological conditions with accompanied impaired body schema, such as phantom limb (Ramachandran, 1998; Ramachandran and Blakeslee, 1998; Ramachandran and Hirstein, 1998) and cerebral palsy (Di Vita et al., 2020).

How can these somatotopagnostic phenomena cause and contribute to the bizarre bodily experiences reported by the illustrative case and the less dramatic reports experienced by other subjects with CRPS? Here we propose two intertwining mechanisms that act together, a bottom-up and a top-down mechanism:

The first mechanism, the Bottom-Up Mechanism, is related to peripheral injury. Impairment to sensory afferent information from the limb can cause cortical reorganization of the primary sensory cortex, leading to impaired bodily representations (Swart et al., 2009; Mancini et al., 2019). This mechanism is not unique to CRPS and is observed in other disorders with primary peripheral damage, such as Phantom Limb, where following limb amputation, cortical sensory representation from neighboring areas of cortex related to the affected limb shift to the deafferented cortical representation (MacIver et al., 2008) and these patients perform slower in body schema handedness tasks (Corradi-Dell’Acqua and Tessari, 2010).

This bodily fragmentation can cause severe distress and anxiety, as suggested by the misrepresentation of the body and the impairment of the topographical continuity of body parts. This was described cleverly in Freud’s Uncanny:

“Dismembered limbs, a severed head, a hand cut off at the wrist, as in a fairy tale of Hauff's, feet which dance by themselves, as in the book by Schaeffer which I mentioned above—all these have something peculiarly uncanny about them, especially when, as in the last instance, they prove capable of independent activity in addition. As we already know, this kind of uncanniness springs from its proximity to the castration complex.” (Freud, 1919, p. 244)

This description directs us to the more speculative second mechanism based on top-down processes. Although Freud connects this uncanny anxiety to the castration complex, it is plausible to think that the origin of this anxiety could be related to various psychological conflicts and not necessarily limited to sexual oedipal conflicts. The cathexis of psychological conflicts onto the affected body part, whether associated with a sexual theme or any other theme, can be explained through a top-down mechanism leading to the projection of mental, psychological ideation and conflicts onto the affected limb. The cortical reorganization and the impaired representations serve as a nidus around which the mental images are cathected. Freud already described this as “Proclivity” (Freud and Breuer, 1895) and “Somatic Compliance” (Freud, 1905[1901]). Freud illustrates this by a metaphor of a grain of dust (the neurological impairment) inside an oyster around which the pearl (the psychosomatic symptom) crystallizes (Freud, 1905[1901]). The traumatic sexual ideations are probably anchored around the bodily deficits and lead to the dramatic presentation described in the clinical vignette. The lack of substantial PTSD symptomatology in the CRPS group could further explain why presentations similar to that of the index patient are very rare.

A more contemporary psychoanalytic formulation is that of René Roussillon, who claims that hallucinatory traumatic perceptions infiltrate the impaired body part (Roussillon, 2011). According to Roussillon, the somatic ailment or somatic affliction is bound by a split-off traumatic state culminating in a state where part of the body is sacrificed to “bind” the threatening psychic representations.

## Limitations

Our study has a few limitations. The first is the small number of subjects. However, the significance of the results even at this number of participants suggests that the results are of true value and not mere chance. The second is the small number of experimental stimuli. However, we preferred to use well-validated stimuli and only stimuli from WAIS-III and WAIS-R. The third is the nature of the experimental stimuli, as the non-human objects were both animate and inanimate. A follow-up study could examine the performance on three conditions—inanimate objects, non-human animate objects, and human animate objects. The fourth is the contribution of pain symptoms to the subjects’ performance with CRPS. As CRPS subjects had relatively severe actual pain during the performance of the experimental task, it might be argued that the pain followed by inattention might have impaired their performance as compared with the control group. However, the pain could not explain the differentiated performance between HBO and Non-HBO stimuli. The fifth is the lack of clinical information in the CRPS subjects regarding psychotic ideation, especially somatic delusions. A similar observation could have strengthened our speculative hypothesis regarding the putative top-down cathexis. The BATH questionnaire sheds some light, although indirectly, on this dimension. Further studies can look directly at psychotic ideations and dissociations in this population in the context of body misrepresentations.

## Conclusion

Patients with pain syndromes in general and CRPS, in particular, can present with what seems *prima facie* to be bizarre and semi-psychotic ideations related to their body. Based on our findings, we argue that these ideations are a combination of concrete cognitive deficit of body structural descriptions with psychological representation and cathexes that can be related to past traumatic experiences. This understanding can enable the clinician to listen to these patients more understandingly. It can also pave the way for further research looking at CRPS as a disease model for somatization and conversion disorders through classic analytic understanding, particularly the concept of somatic compliance.

To conclude, we were able to demonstrate a relative impairment in HBOA in subjects with CRPS compared with controls and a correlation of this task with the clinical characteristic of the patients. Further research can look at the utilization of the HBO task as a marker and as a clinical tool in the assessment of subjects with CRPS, especially the severity of their CRPS symptomatology.

## Data Availability Statement

The raw data supporting the conclusions of this article will be made available by the authors, without undue reservation.

## Ethics Statement

The study was approved by the ethical committee of Chaim Sheba Medical Center and following the Helsinki declaration. All subjects were explained the study protocol and gave written informed consent.

## Author Contributions

IB, AB, LA, and AT – Study design. LA – Data collection. IB, AB, LA, NB, EB, and AT – Data interpretation. IB, AB, and LA – Data analysis. IB – Manuscript drafting. IB, AB, LA, NB, EB, and AT – Critical review. All authors contributed to the article and approved the submitted version.

## Conflict of Interest

The authors declare that the research was conducted in the absence of any commercial or financial relationships that could be construed as a potential conflict of interest.

## Publisher’s Note

All claims expressed in this article are solely those of the authors and do not necessarily represent those of their affiliated organizations, or those of the publisher, the editors and the reviewers. Any product that may be evaluated in this article, or claim that may be made by its manufacturer, is not guaranteed or endorsed by the publisher.
